# Study on tinnitus-related electroencephalogram microstates in patients with vestibular schwannomas

**DOI:** 10.3389/fnins.2023.1159019

**Published:** 2023-04-06

**Authors:** Chi Zhang, Xiaoguang Wang, Zhiwei Ding, Hanwen Zhou, Peng Liu, Xinmiao Xue, Li Wang, Yuke Jiang, Jiyue Chen, Weidong Shen, Shiming Yang, Fangyuan Wang

**Affiliations:** ^1^The First Medical Center, Chinese PLA General Hospital, Beijing, China; ^2^Zhan Tan Temple Outpatient Department, Central Medical Branch of PLA General Hospital, Beijing, China; ^3^College of Otolaryngology Head and Neck Surgery, Chinese PLA General Hospital, Beijing, China; ^4^Medical School of Chinese PLA, Beijing, China; ^5^The Sixth Medical Center, Chinese PLA General Hospital, Beijing, China

**Keywords:** tinnitus, vestibular schwannoma, EEG, microstates, THI

## Abstract

Tinnitus is closely associated with cognition functioning. In order to clarify the central reorganization of tinnitus in patients with vestibular schwannoma (*VS*), this study explored the aberrant dynamics of electroencephalogram (EEG) microstates and their correlations with tinnitus features in *VS* patients. Clinical and EEG data were collected from 98 *VS* patients, including 76 with tinnitus and 22 without tinnitus. Microstates were clustered into four categories. Our EEG microstate analysis revealed that *VS* patients with tinnitus exhibited an increased frequency of microstate C compared to those without tinnitus. Furthermore, correlation analysis demonstrated that the Tinnitus Handicap Inventory (THI) score was negatively associated with the duration of microstate A and positively associated with the frequency of microstate C. These findings suggest that the time series and syntax characteristics of EEG microstates differ significantly between *VS* patients with and without tinnitus, potentially reflecting abnormal allocation of neural resources and transition of functional brain activity. Our results provide a foundation for developing diverse treatments for tinnitus in *VS* patients.

## Introduction

Tinnitus is defined as the perception of sound without external stimulation, and its high incidence results in a significant social and economic burden (Stockdale et al., 2017). Tinnitus is closely associated with cognition and central reorganization (Mohamad et al., 2016). However, the findings of tinnitus-related studies to date have been inconsistent, which may be attributed to the high heterogeneity of characteristics among their populations and the presence of various comorbidities. Therefore, conducting studies with a low degree of heterogeneity is necessary to obtain reliable results (Landgrebe et al., 2010).

Vestibular schwannoma is the most common tumor to occur in the cerebellopontine angle area (Rahimpour et al., 2016). It has been estimated that the disease affects 1.09 out of every 100,000 people in the United States and 2.55–3.32 out of every 100,000 people in Holland (Kleijwegt et al., 2016). Similar to noise-induced tinnitus, tinnitus experienced by *VS* patients is influenced by auditory system interference factors and has fewer confounding factors. Additionally, it has been demonstrated that vestibular schwannoma-induced tinnitus is not significantly correlated with gender, age, tumor size, or hearing status (Kohno et al., 2014). Therefore, vestibular schwannoma-induced tinnitus may serve as a suitable model for overcoming heterogeneity in relevant studies.

EEG allows for the recording of the brain’s electrical activity through scalp electrodes. It reflects various neurocognitive functions and internal brain activities that can significantly contribute to understanding the potential mechanisms of neuronal interactions related to different diseases (Roth et al., 2014; Vasicek et al., 2014). EEG is increasingly being used for detecting tinnitus (Sadeghijam et al., 2021; Zhang et al., 2021). Tinnitus is particularly prominent in a quiet environment. Therefore, evaluating patients’ quiescent EEG in a calm environment is an effective means of studying tinnitus-related abnormal brain electrophysiological activities (Schlee et al., 2014). Classical EEG studies mainly focus on different frequency bands, namely alpha (8–12 Hz), beta (12–30 Hz), theta (4–7 Hz), delta (1–3 Hz), and gamma (30–70 Hz) frequency bands. However, the study of EEG in single frequency band has some limitations (Newson and Thiagarajan, 2018). Evidence indicates that tinnitus represents a change in the attributes of the whole brain network (De Ridder et al., 2014). Besides, local brain responses do not fully represent tinnitus characteristics. Therefore, an indicator system that can measure tinnitus from the whole brain is needed to fully comprehend these characteristics.

EEG microstates are a topology-based method that captures the time-varying changes in global EEG features associated with a specific condition. As a novel EEG research method, EEG microstates measure relatively stable spatiotemporal activities of the brain as they remain steady for specific times (60–120 ms) and then rapidly switch to another relatively stable state (Lehmann, 2010). Indeed, they can be used as the primary construction modalities of the conscious process (Liu et al., 2020), since they can measure changes in the brain network state from global attributes. In psychology, psychological activities and cognitive processing should be a series of transient and relatively stable states. EEG microstates reflect the relatively stable spatiotemporal activities in the brain consciousness perception (Michel and Koenig, 2018). The time course of the microstates represents the rapid switching of different neuronal components in the brain. Moreover, its rich grammatical knowledge also provides a variety of quantitative methods for analyzing EEG data, and thus has essential neurophysiological significance (Khanna et al., 2015). EEG microstates research has been widely applied in neuroscience, cognitive psychology, psychophysiology research, and the diagnosis of various brain diseases, such as Alzheimer’s disease, child hyperactivity disorder, epilepsy, and sleep disorders (Mishra et al., 2020).

Tinnitus-based studies on EEG microstates have also been reported in some studies (Cai et al., 2018, 2019; Cao et al., 2020). Nevertheless, no studies on EEG microstates of *VS* patients with tinnitus have been reported. Higher incidence of tinnitus in *VS* patients indicates that the mechanism of tinnitus may be different from that in healthy people (Leong and Lesser, 2015). And surgical treatment has no or unpredictable effects on tinnitus for *VS* patients (Goldbrunner et al., 2020). Therefore, we intend to explore tinnitus-associated biomarkers in *VS* patients through EEG microstates research, and provide insights for developing personalized treatment approaches for tinnitus in these patients and potentially improve treatment outcomes and prognosis.

## Materials and methods

### Clinical data collection

A total of 146 cases of vestibular schwannoma were enrolled from the outpatient and inpatient departments of the PLA General Hospital. The collected clinical information included age, gender, patient medical history, tumor side, tumor size, pure tone averages (PTAs), commonly used hand, education background, tinnitus period, loudness, and SAS, SDS VAS, and THI scores. This study was performed in line with the principles of the Declaration of Helsinki. Approval was granted by the Ethics Committee of Chinese PLA General Hospital (No. S2021-179-02).

**Figure 1:**
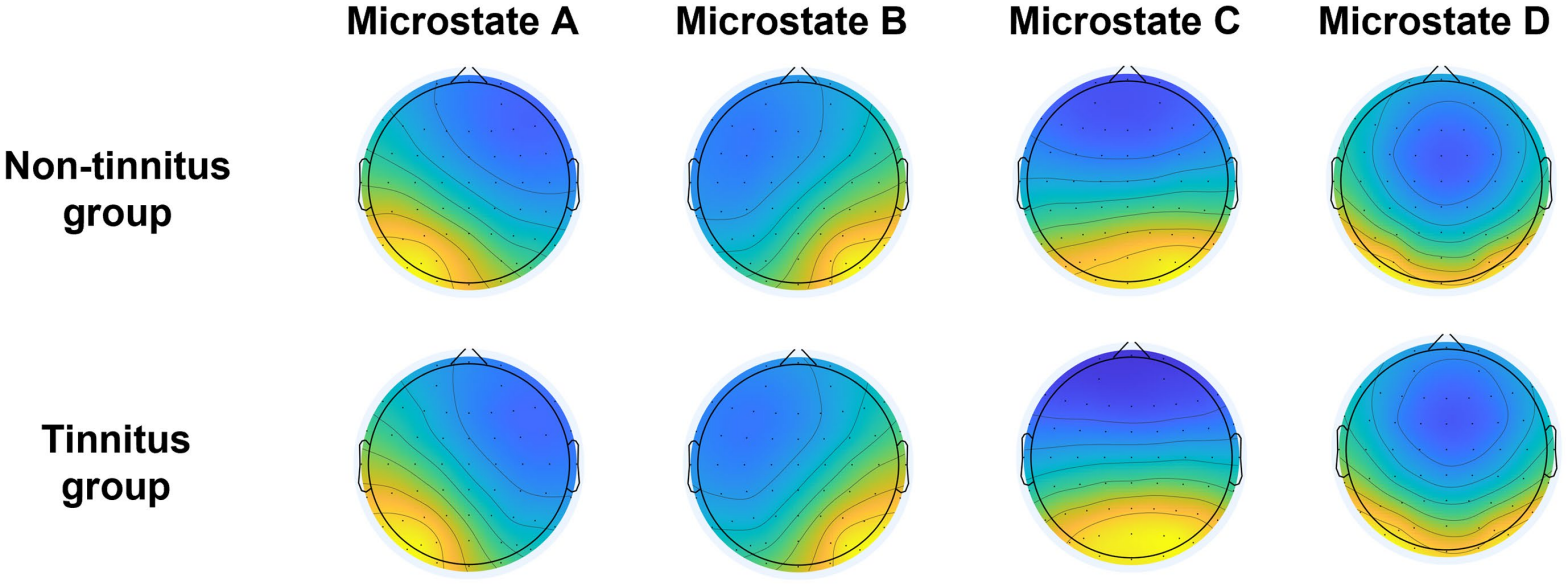
Topological maps of clustering microstates of the non-tinnitus group and the tinnitus group.

Exclusion criteria included: (1) patients with severe anxiety or depression diagnosed by SAS and SDS scales, (2) patients with neurofibromatosis type II, (3) non-right-handed patients, (4) accompanied with severe other intracranial organic diseases, and (5) and having a history of psychotropic drug use or head injury in the last 3 months. Accordingly, a total of 106 patients were enrolled (Figure 1).

### Collection of EEG data

Entire process of EEG data collection was conducted in a soundproof room under electric shielding. The temperature was maintained at 28°C, and outdoor personnel was prevented from walking to ensure that subjects were in a good mental state. Before collecting EEG data, we also considered removing skin cutin, using Quik-Gel conductive gel to fill the space between the electrode and scalp, and controlling all scalp resistance below 10 kΩ. During the data collection process, we told the subjects to close their eyes, relax, and keep still and awake. The whole quiescent EEG data sampling time were about 10–15 min. The EEG data were collected using a 64-guide EEG recorder (Neuroscan). The EEG recording system is a Curry 8 system with a data sampling rate of 1,000 Hz.

### Pre-processing of EEG data

EEG data were pre-processed using EEGLAB (v13.0.0) in MATLAB (R2013b) following some steps that were conducted according to international practices (Jia and Yu, 2019). (1) EEG data were imported, and all electrodes were repositioned. (2) Useless electrodes were removed within the EEG records. (3) The filtering range was 0.1–70 Hz, and the concave filter of 48–52 Hz was used for processing. (4) Whole brain average re-reference was performed. (5) We browsed the data, manually removed that with significant drift and artifacts, and recorded the bad electrode. (6) Spherical spline interpolation was also used for the channels with poor records. (7) We further performed independent component analysis (ICA), analyzed various independent components, and excluded them by checking the corresponding independent components of muscle artifacts, eye movement, and heart beats. (8) We finally segmented the recorded EEG data and considered 2000 ms as a unit (Lan et al., 2021). (9) For the whole brain electrode, we deleted the segments whose amplitude exceeds ±80 μV in all channel records. Subjects with significant drift exceeding 20% of the recording time or subjects with more than five defective electrodes shall would be excluded. Therefore, 8 cases with EEG-defective information were excluded. Finally, 22 cases of vestibular schwannoma without tinnitus and 76 cases of vestibular schwannoma with tinnitus were enrolled in the final analysis of this study (Figure 1). The mean number of segments was 314 in the tinnitus group and 317 in the non-tinnitus group.

### EEG microstates calculation and indicators

The calculation method of EEG microstates was conducted according to the standard process that were reported in previous studies (Cai et al., 2018). First, the pre-processed EEG segment data were filtered by 2–20 Hz band-pass filtering. This band-pass filter was used as a consequence of previous studies (Nishida et al., 2013), into the nature of microstates recorded in a multichannel array over the scalp and the alpha frequency band (8–12 Hz) of the multichannel resting-state EEG signal. Then, the global field power (GFP), which reflects the change of potential across multiple electrodes at different time points (i.e., the overall intensity of brain electric field at other time points), was calculated by the following equation:

$$\mathrm{GFP}\left(t\right)=\sqrt{\frac{1}{K}\overset{K}{\underset{i=1}{\sum }}{\left(V_{i}\left(t\right)-V_{\mathrm{mean}}\left(t\right)\right)}^{2}}$$

wherein K is the number of electrodes, V_i_ is the transient potential of the *i*th electrode at moment *t*, and V_mean_ (t) is the average transient potential of all electrodes at moment *t*. GFP is usually used to reflect the rapid changes in brain activity. Many peaks and troughs were expected in the whole EEG recording process. The peak topological map had a better signal-to-noise ratio and was more similar to the topological map at the nearby time point. The valley topological map was quite different from the one at the nearby time point, which was considered a switching process for different microstates. We extracted the topological maps of EEG at the local maximum of the GFP curve as the representative topological map. Based on the Atomize and Agglomerate Hierarchical Clustering (AAHC) algorithm, we performed clustering analysis and divided the usual topological maps of EEG into microstates A, B, C, and D (Khanna et al., 2015). Each of the group subsets is assigned to the same class as the grand subset that this group subset shows most similarity. After classifying the topological maps of all subjects, we calculated the microstates parameters, including the duration of the microstate (the length of time for microstate to remain stable), frequency (the average frequency of a certain type per second), and coverage (the percentage of time records dominated by microstates with non-random conversion probability).

### Statistical analysis

All data were statistically analyzed by SPSS26.0 software. Measurement data subject to normal distribution were expressed as mean ± standard deviation. In contrast, counting data not conforming to normal distribution were expressed by median (interquartile interval). The independent sample t-test was employed for group comparisons of count data that conform to a normal distribution, while the Mann–Whitney *U* test was used for count data that deviate from a normal distribution. The Chi-squared test was utilized for comparing quantitative data. Microstates parameters were calculated through the Microstate 0.3 toolkit in MATLAB and debugged and ran according to the script. The intergroup comparison of microstates was performed by independent sample *t*-test with a statistical threshold of *p* < 0.0125, and the correlation analysis of microstates parameters with clinical data was performed by Pearson correlation analysis.

## Result

### Clinical features of patients

We enrolled 22 patients with vestibular schwannoma (*VS*) who did not have tinnitus, with an average age of 46.8 years, including 13 males and 9 females. Among them, 14 had left-sided tumors and 8 had right-sided tumors, with an average tumor size of 19.9 mm. The average hearing level on the side with the tumor was 60.5 dB HL, while the average hearing level on the contralateral side was 15.0 dB HL. Of these patients, 11 reported experiencing vestibular symptoms, while the other 11 did not. Additionally, we enrolled 76 patients with *VS* and tinnitus, with an average age of 47.0 years, including 35 males and 41 females. Among them, 36 had left-sided tumors and 40 had right-sided tumors, with an average tumor size of 17.9 mm. The average hearing level on the side with the tumor was 58.5 dB HL, while the average hearing level on the contralateral side was 16.3 dB HL. Of these patients, 33 reported experiencing vestibular symptoms, while the other 43 did not. There were no significant differences between the two groups in terms of age, gender, tumor side, tumor size, hearing level on the tumor side, hearing level on the contralateral side, vestibular symptoms, or education background (*p*-values = 0.954, 0.281, 0.179, 0.257, 0.775, 0.531, 0.585, and 0.419, respectively) (Table 1). Furthermore, patients with tinnitus reported an average tinnitus duration of 12 months, a loudness of 46.8 dB HL, and scores of 30.7, 41.5, 43.2, and 4.0 on the THI, SAS, SDS, and VAS scales, respectively.

**Table 1:** Clinical characteristics of vestibular schwannoma patients.

Clinical features	Non-tinnitus group	Tinnitus group	*p*-Value
Age (year)	46.8 ± 11.4	47.0 ± 11.1	0.954
Gender (male/female)	13/9	35/41	0.281
Tumor side (left/right)	14/8	36/40	0.179
Tumor size (mm)	19.9 ± 5.8	17.9 ± 7.4	0.257
PTAs on tumor side (dB HL)	60.5 ± 31.8	58.5 ± 27.0	0.775
PTAs on contralateral side (dB HL)	15.0(10.9)	16.3(13.4)	0.531
Vestibular symptoms (yes/no)	11/11	33/43	0.585
Educational background	2/13/7	6/34/36	0.419
Tinnitus loudness (dB HL)	NA	46.8 ± 21.6	NA
Tinnitus duration (month)	NA	12(57)	NA
THI score	NA	30.7 ± 21.5	NA
SAS score	NA	41.5 ± 9.4	NA
SDS score	NA	43.2 ± 10.1	NA
VAS score	NA	4.0 ± 1.9	NA

PTAs, pure tone averages; Educational background, including primary education, secondary Education and Bachelor degree or above; THI, tinnitus handicap inventory; SAS, self-rating depression scale; SDS, self-rating anxiety scale; VAS, visual analog scale; NA, not applicable.

### Microstate analysis

**Figure 2:**
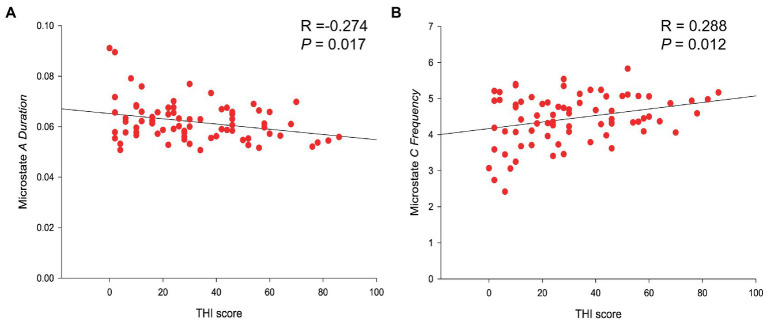
Linear regression results of microstate parameters and THI score. **(A)** THI score was negatively related to the duration of microstates A (*R*-value = −0.274; *p* -value = 0.017). **(B)** THI score was positively correlated with the frequency of microstate C (*R* -value = 0.288; *p* -value = 0.012).

After hierarchical clustering of non-tinnitus group and tinnitus group, two groups of microstates were constructed, including A, B, C, and D, respectively. The clustering topological map phenotype was consistent with previous literature findings (Figure 2). We identified the topological map of each patient and matched it with the microstates across groups. The average global explanation variances of the non-tinnitus and the tinnitus groups were 77.5 and 78.0%, respectively.

The microstates results showed that the frequency of microstates C of the non-tinnitus group was significantly lower than that in the tinnitus group (4.040 versus 4.452 times/s; *p*-value = 0.010) (Table 2). The statistical results passed the multiple comparison correction. In addition, syntax analysis showed that the transition probability of the non-tinnitus group from microstates D to microstates C was lower than that of the tinnitus one (0.078 versus 0.091; *p*-value = 0.024), although not pass the multiple comparison correction. No significant difference was found in other indicators. We also investigated the differences in microstate indicators between *VS* patients with left and right tumors, but no statistically significant differences were found.

**Table 2:** Parameters of the non-tinnitus group and the tinnitus group.

	Non-tinnitus group		Tinnitus group		
Microstates Parameter	Mean	SD	Mean	SD	*p*-Value
Duration (s)					
Microstate A	0.064	0.007	0.062	0.008	0.223
Microstate B	0.065	0.009	0.062	0.011	0.260
Microstate C	0.065	0.013	0.066	0.015	0.819
Microstate D	0.063	0.013	0.059	0.013	0.248
Frequency (times/s)					
Microstate A	3.921	0.906	3.964	0.980	0.853
Microstate B	4.026	0.682	4.050	0.875	0.904
Microstate C	4.040	0.534	4.452	0.678	0.010^*^
Microstate D	3.735	0.784	3.844	0.668	0.518
Coverage					
Microstate A	0.250	0.065	0.242	0.064	0.640
Microstate B	0.256	0.059	0.246	0.064	0.483
Microstate C	0.261	0.068	0.286	0.071	0.142
Microstate D	0.233	0.077	0.226	0.064	0.655

SD, standard deviation, ^*^p < 0.05.

### Correlation analysis

**Figure 3:**
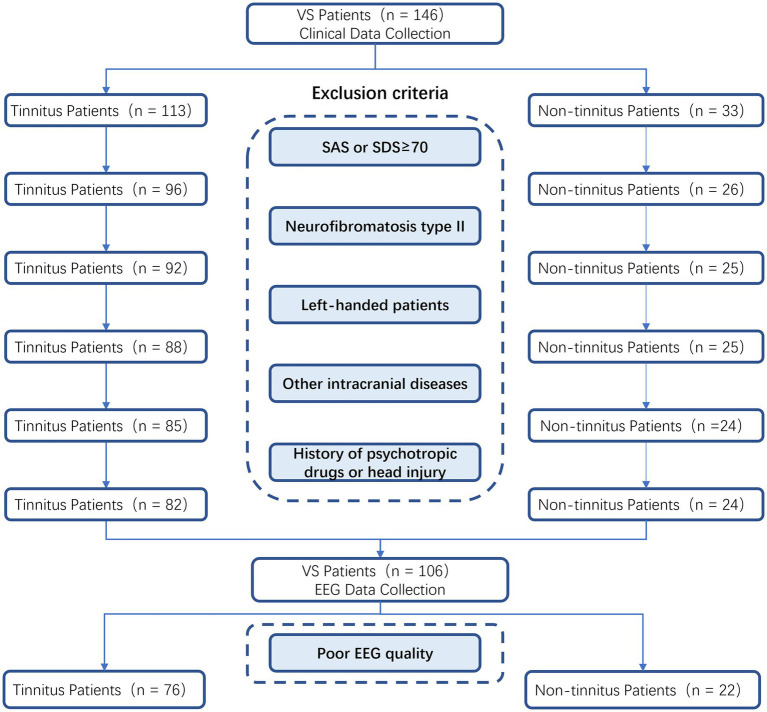
Flowchart for screening clinical and EEG data.

Correlation analysis was conducted on the clinical data related to tinnitus of *VS* patients, including tinnitus loudness and duration, THI and VAS scores, and microstates parameters (duration, frequency, coverage, and probability of transition). It was found that the THI score was negatively related to the duration of microstates A (*R*-value = −0.274; *p*-value = 0.017), positively correlated with the frequency of microstate C (*R*-value = 0.288; *p*-value = 0.012) (Table 3 and Figure 3). No significant correlations were found in the changes of microstate features with other clinical data in the tinnitus group.

**Table 3:** Correlation analysis of THI scores with microstates duration, frequency, and coverage.

Microstates category		Duration	Frequency	Coverage
Microstate A	*R*-value	−0.274	0.148	−0.001
	*P*-value	0.017^*^	0.202	0.996
Microstate B	*R*-value	−0.176	0.205	0.037
	*P*-value	0.128	0.076	0.753
Microstate C	*R*-value	−0.133	0.288	0.038
	*P*-value	0.253	0.012^*^	0.743
Microstate D	*R*-value	−0.174	0.115	−0.077
	*P*-value	0.132	0.324	0.510

^*^p < 0.05.

## Discussion

To our knowledge, this is the first time to study the abnormal dynamics of EEG microstates in *VS* patients with tinnitus. There is evidence that the tinnitus perception is related to cognition and cortical plastic changes. Meanwhile, EEG research has been widely used in the field of cognition (Cecchetti et al., 2022). The purpose of this study was to evaluate cortical plasticity in *VS* patients with tinnitus by using EEG microstate. There is a significant difference in the temporal characteristics of EEG microstate between non-tinnitus group and tinnitus group.

In this study, the THI scale, which is commonly accepted in the guidelines of various countries, was selected as the relatively objective evaluation index of tinnitus (Cima et al., 2019). There is no significant statistical difference in clinical indicators between the tinnitus group and the non-tinnitus group, which can effectively avoid the impact of clinical indicators on EEG microstate. This study excluded normal individuals with tinnitus to avoid confounding factors that could affect EEG signals. As tumors can potentially affect EEG signals, and patients with vestibular schwannoma typically experience severe and prolonged hearing loss which can result in aberrant brain network activity compared to those with typical tinnitus.

EEG microstates were divided into four categories, and the average global explanation variance in the none-tinnitus group and tinnitus group were 77.5 and 78.0%, respectively. Currently, the best number of clusters of EEG microstates that are widely used is four, and the correlation between microstates and quiescent functional magnetic resonance imaging has been confirmed (Britz et al., 2010; Musso et al., 2010; Van de Ville et al., 2010). These four clusters can explain 65 to 84% of the global explanation variance (Brodbeck et al., 2012; Tomescu et al., 2014) and have a high degree of similarity in different studies (Gao et al., 2017). Our results are consistent with previous studies, providing convincing evidence. Previous research has shown that microstate A is primarily associated with auditory and speech processing, while microstate B is associated with processing extrinsic visual information. Microstate C corresponds to the salience network, while microstate D primarily reflects the attention network (Michel and Koenig, 2018).

Among *VS* patients with tinnitus in this study, microstates C increased in frequency, and the frequency of microstates C showed a significant positive correlation with the THI score. According to a 15-year meta-analysis on microstates in schizophrenia, microstate C is more frequently observed in patients with schizophrenia (Rieger et al., 2016). Furthermore, studies suggest that the frequency of microstate C is significantly increased and positively correlated with hallucination perception (Palaniyappan and Liddle, 2012). Thus, tinnitus, as a positive illusion, may be linked to this phenomenon. Additionally, studies have shown that microstate C is primarily related to the positive blood oxygen level-dependent signal activation in the posterior–anterior cingulate gyrus, bilateral lower frontal lobes, and right forebrain islands, representing a salience network (Seeley et al., 2007; Taylor et al., 2009; Lin et al., 2022), which can detect and locate changes in the neural networks caused by various endogenous and exogenous stimuli, and can also integrate stimuli from the auditory pathway (Heywood et al., 2017). Evidence suggests that tinnitus patients exhibit abnormal functioning in the salience network, as demonstrated by investigations using functional magnetic resonance imaging (Amaral and Langers, 2015). This disruption of the salience network can interfere with connections between the default and central executive networks, leading to cognitive dysfunction in these patients. These disruptions have been related to some behavioral performances of tinnitus patients, such as attention, anti-interference ability, and executive function abnormalities (Hallam et al., 2004; Stevens et al., 2007; Araneda et al., 2015). Interestingly, previous studies on tinnitus microstates have not found significant differences between tinnitus patients and healthy person (Cai et al., 2018). However, tinnitus patients did exhibit an increase in the frequency of microstate C. It is worth noting that *VS* patients have been found to have functional abnormalities in memory and information processing speed (Deng et al., 2022), which are closely related to the salience network. Furthermore, individuals with *VS* tend to have severe hearing loss, which result in abnormal functional connectivity with the salience network (Xu et al., 2019). These factors may work together to cause abnormalities in microstate C between *VS* tinnitus patients and *VS* non-tinnitus patients.

Correlation analysis demonstrated a negative correlation between the THI score and the duration of microstates A. Britz et al. (2010) proved, through simultaneous EEG and functional magnetic resonance imaging, that microstates A is related to the activation degree of the bilateral superior temporal gyrus and the middle temporal gyrus and is associated with the hearing and speech processing of patients. It has been shown that pathological reorganization occurs in the temporal region and auditory cortex of patients with tinnitus (Chen et al., 2016, 2017). Accordingly, the abnormality of microstates A might strongly suggest that more significant changes in the auditory network have undergone to *VS* patients accompanied by tinnitus than others without tinnitus. In addition, some studies suggest that microstate A may be linked to the default network (Drissi et al., 2016), while the salience network can effectively suppress the default network (Menon et al., 2023). From this perspective, it is not unexpected for microstate A and microstate C to display contrasting patterns. While a negative correlation between the duration of microstate A and the THI score was not found in previous studies of idiopathic sudden sensorineural hearing loss patients with tinnitus (Cai et al., 2019), they did find that the duration of microstate A in tinnitus patients was significantly reduced, which is similar with our study.

In contrast to the abnormality of microstate D observed in normal individuals with tinnitus (Cai et al., 2018), we did not detect any differences in *VS* patients with tinnitus compared to those without tinnitus. Microstate D characterizes dorsal attention network, encompassing the right superior frontal gyrus, middle frontal gyrus, and right superior and inferior parietal lobules (Britz et al., 2010). Microstate D reflects real-time updates of endogenous neural activity and is associated with the switching and reorientation of attention (Faber et al., 2017). Higher levels of microstate D correspond to greater alertness (Wingelaar-Jagt et al., 2021). *VS* patients exhibited significant attentional impairments (Deng et al., 2022), which may have masked potential microstate D abnormalities observed in normal tinnitus patients. Our study also found no significant differences in microstate class B between the two groups, which aligns with previous research on tinnitus microstates. According to previous studies, microstate class B is related to activities in the visual network, including the bilateral lateral extrastriate visual areas (Michel and Koenig, 2018). These findings suggest that the visual network in *VS* patients with tinnitus may not be significantly damaged when compared to non-tinnitus *VS* patients.

To sum up, our results show that the large-scale brain functional network of *VS* patients with tinnitus has abnormal changes compared with those without tinnitus, especially in the salience network. These findings provide a theoretical basis for the diversified treatment of *VS* patients with tinnitus, as surgery can not relieve tinnitus effectively in all *VS* patients. For instance, noninvasive techniques such as repetitive transcranial magnetic stimulation (rTMS) can modulate the excitability of the brain cortex (Formánek et al., 2018), and our results suggest that abnormal central networks could be potential targets for tinnitus treatment in *VS* patients.

This study has several limitations that should be noted. The sample size of EEG data collected from *VS* patients was limited by the very low incidence of vestibular schwannoma (2.55–3.32/100,000) (Kleijwegt et al., 2016). As a result, our subgroup comparisons were limited to tumor side classification, and we were unable to conduct more detailed and specific analysis. Therefore, future studies with larger sample sizes are needed to conduct more in-depth research. Despite these limitations, our study provides valuable insights into the neurophysiological mechanisms underlying tinnitus in *VS* patients.

## Data availability statement

The raw data supporting the conclusions of this article will be made available by the authors, without undue reservation.

## Ethics statement

The studies involving human participants were reviewed and approved by the Ethics Committee of Chinese PLA General Hospital. The patients/participants provided their written informed consent to participate in this study.

## Author contributions

CZ, XW, and FW: study design. CZ, ZD, HZ, PL, and XX: data collection. CZ, XW, and ZD: data analysis and manuscript drafting. FW, SY, WS, JC, and FW: recruitment of patients. All authors contributed to the article and approved the submitted version.

## Funding

This work was supported by grants from the National Key Research and Development Project (2019YFC0121302); the National Key Research and Development Project (2019YFC0840707); the Beijing Nova Program (Z201100006820133); the National Key Research and Development Project (2020YFC2005203); and the National Natural Science Foundation of China (81820108009).

## Conflict of interest

The authors declare that the research was conducted in the absence of any commercial or financial relationships that could be construed as a potential conflict of interest.

## Publisher’s note

All claims expressed in this article are solely those of the authors and do not necessarily represent those of their affiliated organizations, or those of the publisher, the editors and the reviewers. Any product that may be evaluated in this article, or claim that may be made by its manufacturer, is not guaranteed or endorsed by the publisher.
